# Effects of an internet-based combined exercise and cognitive-behavioral therapy intervention on endocannabinoid system biomarkers and physical fitness in adults with mild-to-moderate depression: a SONRIE randomized controlled trial

**DOI:** 10.1007/s00406-026-02330-x

**Published:** 2026-08-04

**Authors:** Manuel Ruiz-Muñoz, David Jiménez-Pavón, Inmaculada Garrido-Palomino, Juan Manuel Escudier-Vázquez, Eulalio Juan Valmisa Gómez de Lara, Vanesa España-Romero

**Affiliations:** 1 MOVE-IT Research group, Department of Physical Education, Faculty of Education Sciences, University of Cádiz, Puerto Real, Cádiz, Spain; 2 Biomedical Research and Innovation Institute of Cadiz (INiBICA), Puerta del Mar University Hospital, University of Cádiz, Cádiz, Spain; 3 Mental Health Service, Puerto Real University Hospital, Puerto Real, Cádiz, Spain; 4 CIBER of Frailty and Healthy Aging (CIBERFES), Instituto de Salud Carlos III, Madrid, Spain; 5 Department of Psychology, Sociology and Philosophy, Faculty of Education, University of León, León, Spain; 6 C-HIPPER Climbing Research Association, Cádiz, Spain

**Keywords:** Depression, Physical exercise, Cognitive behavioral therapy, Internet-based interventions, Cardiorespiratory fitness, Muscular strength, Endocannabinoid system, 2-Arachidonoylglycerol, Anandamide, Randomized controlled trial

## Abstract

**Background:**

This SONRIE randomized controlled trial (NCT05849792) examined the effects of a 12-week combined physical exercise and internet-based cognitive-behavioral therapy (iCBT) intervention on endocannabinoid system (ES) biomarkers and physical fitness in adults with mild-to-moderate depression.

**Methods:**

Eighty adults were randomly assigned 1:1 to an intervention (IG) or control (CG) group. Outcomes included nine ES biomarkers (2-arachidonoylglycerol, 2-AG; anandamide, AEA; seven analogues) and physical fitness, including cardiorespiratory fitness (CRF; 6-minute walking test) and muscular strength. Measurements were taken at baseline, post-intervention and 8-week follow-up. Primary analysis performed 2 × 3 repeated-measures ANOVA on completers; mixed-effects intention-to-treat model as sensitivity analysis.

**Results:**

No significant time × group interaction was detected for any ES biomarker, including 2-AG (F(2,88) = 0.17, *p* = 0.844) and AEA (F(2,88) = 1.20, *p* = 0.306); both groups showed comparable within-group decreases in 2-AG, 2-LG and 2-OG. The intervention significantly improved CRF [between-group difference + 81.6 m at 12 weeks (F(2,78) = 7.88, *p* = 0.001)], exceeding the established minimal clinically important difference. None of the exploratory muscular fitness outcomes reached statistical significance; the arm curl test showed a borderline non-significant interaction (F(2,78) = 2.83, *p* = 0.065).

**Conclusion:**

A 12-week internet-based combined exercise and iCBT intervention significantly improved CRF in adults with mild-to-moderate depression. We did not find evidence of an intervention-specific effect on plasma ES biomarkers. These findings support the inclusion of internet-delivered exercise and psychological interventions in comprehensive treatment strategies for depression.

**Trial registration:**

ClinicalTrials.gov NCT05849792 (registered 6 May 2023).

**Supplementary Information:**

The online version contains supplementary material available at https://doi.org/10.1007/s00406-026-02330-x.

## Introduction

Emerging evidence implicates the endocannabinoid system (ES) in modulating both physiological and psychological health, influencing pathways relevant to depression and other disorders [26, 32]. The ES includes two main endocannabinoids, anandamide (AEA) and 2-arachidonoylglycerol (2-AG), released from the postsynaptic neuron and traveling back to the presynaptic neuron to modulate neurotransmitter release [26]. Analogues such as palmitoylethanolamide (PEA) and oleoylethanolamide (OEA) interact with the system through different mechanisms [26]. While AEA and 2-AG exhibit varying affinities for cannabinoid receptors (CB-1 and CB-2), PEA and OEA do not bind directly to these receptors; instead, PEA acts through peroxisome proliferator-activated receptors, and OEA interacts with G protein-coupled receptors [32, 53]. This network regulates inflammatory and immune responses, modulating cytokine production and immune cell function [53, 62]. Additional ES biomarkers — 2-linoleoylglycerol (2-LG), 2-oleoylglycerol (2-OG), docosatetraenoylethanolamide (DEA), docosahexaenoylethanolamide (DHEA), linoleylethanolamine (LEA) and stearoylethanolamine (SEA) — also play roles in cognitive and emotional behaviors, neurogenesis and neurotrophin levels, often dysregulated in depressive disorders [10, 34, 74].

Physical exercise is well documented to improve overall physical and mental well-being [23, 40]. In particular, multiple randomized trials and meta-analyses have demonstrated that supervised aerobic and resistance exercise programs reduce depressive symptoms in patients with major depressive disorder [47, 63], with effect sizes comparable to first-line pharmacological treatments. Despite this evidence, exercise prescription remains underused in routine psychiatric care [67], partly because illness-related symptoms and access barriers can make structured exercise difficult to sustain. In depressive populations, illness-related symptoms may also modulate the ES, while both acute and chronic exercise have been shown to influence ES ligands [19, 30, 43, 69, 71]. Acute bouts of exercise elevate these ES ligands, a change that persists into the recovery period [10, 29, 37, 54, 73]. However, the chronic effects of sustained exercise on ES ligands present a more complex scenario, with studies indicating variable impacts on ES dynamics [20]. Regular exercise has been posited to lead to sustained modifications in ES concentrations, paralleling improvements in physical fitness indicators such as cardiorespiratory fitness (CRF) and muscular strength [13, 38]. iCBT has been widely acknowledged for its efficacy in managing depressive disorders [39], offering not only acute relief but also sustained benefits that support relapse prevention [6, 18]. While the direct impact of iCBT on the ES is less understood, its potential synergistic effects with physical exercise on physiological health remain underexplored.

In the SONRIE randomized controlled trial [22], we tested the hypothesis that a 12-week combined intervention of internet-delivered physical exercise and iCBT would (i) modify circulating ES biomarkers — primary outcomes 2-AG and AEA — and (ii) improve CRF (primary fitness outcome) in adults with mild-to-moderate depression, compared with treatment as usual. We further hypothesized that these effects would persist during an 8-week follow-up period without intervention. Remaining ES biomarkers (OEA, 2-LG, 2-OG, DEA, DHEA, LEA, SEA) and muscular fitness outcomes (handgrip, arm curl, chair stand, standing jump) were analyzed as exploratory.

## Methods

### Study design

This study is a pre-specified secondary analysis of the SONRIE study, a randomized controlled trial (RCT) registered at ClinicalTrials.gov (Identifier: NCT05849792; registered 6 May 2023). The trial was conducted between September 2020 and December 2023 at the Mental Health Clinical Management Unit of Puerto Real (Cádiz, Spain). It adheres to the 2013 Declaration of Helsinki, and was approved by the Andalusian Biomedical Research Ethics Portal (1875-N-18 CEI/Cádiz). All participants provided written informed consent prior to inclusion. The full trial protocol and statistical analysis plan have been published [22]. The present manuscript reports pre-specified secondary outcomes; deviations from the published statistical analysis plan are detailed in the Statistical Analysis section. The trial is reported in accordance with the CONSORT 2025 statement [33]; a completed checklist is provided as Supplementary Material.

### Patient and public involvement

Patients and members of the public were not involved in the design, conduct or reporting of this trial. The intervention was co-developed by the research team in close collaboration with the clinical staff of the Mental Health Clinical Management Unit, who provided iterative feedback on feasibility and acceptability during pilot stages.

### Eligibility criteria

Eligibility criteria included adults aged 25 to 65 years (highest prevalence of depression in Andalusia; [45]) with a psychiatric diagnosis of mild-to-moderate depression according to International Classification of Diseases 10th Revision (ICD-10) criteria [72]. We limited the study to mild-to-moderate depression to ensure participant safety and maintain a homogeneous sample [63]. Project investigators verified that participants met the remaining criteria, including the ability to engage in physical activity without restrictions. Exclusion criteria included a diagnosis of major depression, the presence of acute or terminal illness, a history of cerebral infarction, epilepsy or brain cancer, and unstable cardiovascular disease or other medical conditions that could interfere with participation in physical exercises. Eligibility criteria for staff delivering the intervention required a degree in Sciences of Physical Activity and Sport with prior experience in clinical exercise programs (exercise specialist) and a doctoral-level qualification in clinical psychology with experience in CBT and emotional regulation (psychologist).

### Participants and randomization

The SONRIE study initially screened 132 individuals from the Mental Health Clinical Management Unit of Puerto Real (Cádiz) through community presentations and social media campaigns. Of these, eighty participants were considered eligible and enrolled in the study.

Participants were randomly assigned 1:1 to the intervention or control group using a computer-generated sequence (STATA 16.0; simple randomization) prepared by the principal investigator before recruitment. Allocations were transmitted to field staff only after each participant’s baseline assessment was completed. Outcome assessors were blinded to group assignment at all time points; data were de-identified before analysis. Participants and personnel delivering the intervention were unblinded, as is unavoidable in non-pharmacological trials.

### Procedure

Participants were instructed to continue their regular lifestyle throughout the study. CG participants received treatment as usual at the Mental Health Clinical Management Unit of Puerto Real. The CG did not receive structured exercise prescription, iCBT or any contact from the research team beyond the three scheduled assessments. CG participants were offered access to the intervention upon completion of the trial. Psychological and health parameters, including ES biomarkers, physical fitness and body composition, were collected at three evaluation points: pre-intervention (baseline), post-intervention (12 weeks later), and retest (8 weeks post-intervention). Each participant attended 3 sessions at each evaluation point. All measurements were performed by trained research staff blinded to group allocation to ensure data accuracy and reliability.

### Physical exercise intervention

This exercise protocol, reported following the Consensus on Exercise Reporting Template (CERT) [64], comprised a 12-week physical exercise regimen with three sessions per week. It included a supervised online session each Tuesday and two unsupervised sessions, featuring balance, resistance and CRF training. Exercise intensity was self-monitored using the Borg Rate of Perceived Exertion (RPE) scale (6–20; [8]), with a target range of 12–14 (“somewhat hard”) for the resistance and CRF components and 11–13 for the balance component. Exercise volume adhered to the World Health Organization’s 2020 physical activity recommendations for adults [70]. Each supervised session was overseen by an exercise specialist with prior experience in clinical populations, supported by a physical therapist during the first four weeks.

Each session started with a 7–10 min dynamic stretch and mobility warm-up, followed by 10 min of progressing balance training, 25–35 min of resistance exercises (squats, lateral and frontal lunges, biceps curl, push-ups, bent-over rows, shoulder press) and 13–14 min of CRF components with dance and step routines. The session concluded with an 8–10 min cool-down incorporating stretching and relaxation techniques. Unsupervised sessions replicated the supervised content through recorded videos. Successful adherence was defined as completing at least 80% of the sessions; achieved adherence in the IG was 72.5% (median 28 of 36 sessions, IQR 24–32).

### Psychological intervention

The psychological component featured an adapted iCBT program, delivered through a secure and encrypted platform, compliant with American Psychological Association guidelines [1]. The iCBT was based on the Unified Protocol for the Transdiagnostic Treatment of Emotional Disorders [2], comprising 18 modules covering physiological, cognitive, emotional and behavioral dimensions. Sessions were video-guided, asynchronous and individually tailored, each lasting no more than 30 min over a 12-week span. Initially, two sessions per week were conducted until week 7, then reduced to one session per week until week 12. The curriculum included psychoeducation, identification and modification of negative cognitions and emotions, interoceptive exercises (breathing techniques and sensory feedback), and behavioral activation. The iCBT program was delivered by a doctoral-level clinical psychologist with experience in CBT and emotional regulation.

### Outcomes

Primary outcomes for the present sub-study were 2-AG and AEA (ES domain) and the 6-minute walking test (CRF domain). Remaining ES biomarkers (OEA, 2-LG, 2-OG, DEA, DHEA, LEA, SEA) and muscular fitness measures (handgrip, arm curl, chair stand, standing jump) were analyzed as exploratory outcomes. The primary contrast for all outcomes was the time × group interaction at the 12-week (post-intervention) time point; the time × group interaction at the 20-week (8-week follow-up) time point was a secondary contrast assessing maintenance of effects.

### Endocannabinoid system biomarkers

Blood samples were collected early morning after an overnight fast, using K2-EDTA tubes to prevent coagulation. Samples were processed immediately to separate plasma by centrifugation at 4 °C. Plasma was analysed for concentrations of AEA, 2-AG, OEA, 2-LG, 2-OG, DEA, DHEA, LEA and SEA using liquid chromatography–tandem mass spectrometry (LC-MS/MS) with electrospray ionization.

### Physical fitness assessments

#### Cardiorespiratory fitness (CRF)

The 6-Minute Walking Test (6MWT) was used to assess CRF. Participants walked as fast as possible for six minutes on a flat, 60-meters circuit [58]. The total distance covered in a single attempt was recorded for further analyses.

#### Muscular strength

Upper-limbs muscle strength was measured using the Arm Curl Test, where participants performed seated arm extension-flexion movements while holding a specified weight (2.3 kg for women and 3.6 kg for men). Each participant completed the test once per arm for 30 s [58]. The number of repetitions was recorded separately for each arm, and the total repetitions from both arms were summed for the analyses. Handgrip strength was also measured using a digital dynamometer (TKK 5101 Grip-D. Participants performed the test sequentially with the right and left hands, adjusting the grip according to the hand size [60]. Each hand was tested twice, and the maximum score archived in kilograms was recorded. The average of the maximum scores from both hands was used for analyses. 

Lower-limbs muscle strength was evaluated using the two tests. In the Chair stand test.

participants were asked to stand up and sit down as many times as possible from a seated position with arms crossed over their chest within a 30-seconds [58]. The total number of repetitions completed was recorded. The Standing long jump test was administered to participants under 45 years of age. They performed two jumps from behind a starting line, jumping forward with feet together as far as possible. The longest distance from the starting line to the heel’s nearest point was measure in centimetres, with the best of two attempts recorded [25].

### Sociodemographic characteristics

Sociodemographic characteristics were collected using a self-designed developed structured questionnaire. The data captured included age, antidepressant usage, economic stress, smoking and alcohol intake, educational level, and marital status. Additionally, information on comorbidities and the impact of bodily pain on daily functioning was obtained by asking whether participants had ever been diagnosed with conditions such as cardiovascular diseases, cancer, or diabetes (recorded as ‘yes’ or ‘no’). Responses for antidepressant usage and economic stress were binary (“yes” or “no” for medication usage; “easy” or “difficult” for economic stress). Smoking status and alcohol intake were classified into three categories: “current”, “former” or “never”. Educational level was dichotomized into “up to primary education” or “higher education”, and marital status was coded as “single” or “in a relationship”. The persistence of depression was recorded as the number of years since the diagnosis. The impact of bodily pain on daily functioning was assessed using a specific question from the 36-Item Short Form Survey regarding pain interference with work, with responses categorized from “no to moderately” to “quite to extremely”.

### Body composition

Body composition was measured using a multifrequency bioimpedance analyzer (TANITA-MC780MA). Body weight, body mass index (BMI), and fat mass percentage were recorded. Height was measured in the Frankfurt plane using a stadiometer (Type SECA 225).

### Harms

Adverse events related to the exercise intervention were assessed non-systematically: participants were instructed to report any musculoskeletal pain, dizziness or other discomfort during or after sessions to the exercise specialist, who maintained a log of incidents. No serious adverse events related to the intervention were reported. Minor self-limited muscle soreness during the first two weeks was reported by 6 (15%) IG participants. No adverse events were reported in the CG.

### Sample size

The original SONRIE trial sample size of 80 participants (40 per group) was determined to detect a between-group mean difference of 8.26 points on the Beck Depression Inventory (the primary outcome of the trial) with 80% power and α = 0.05, two-sided [22]. The present manuscript reports pre-specified secondary outcomes from the same trial. Post-hoc estimation indicates that the achieved analytic samples (*n* = 44 for ES biomarkers; *n* = 39 for the 6MWT) provided 80% power to detect between-group standardized mean differences of approximately Cohen’s d = 0.85 for ES outcomes and d = 0.95 for CRF at the 12-week time point.

### Statistical analysis

Distributions were assessed by visual inspection of frequency histograms and Shapiro–Wilk tests; homogeneity of variances was assessed using Breusch–Pagan tests [11]. Participant characteristics are presented as means ± standard deviations (SDs) for continuous variables and frequencies (%) for categorical variables. Differences between groups at baseline were tested with one-way ANOVA for continuous variables and chi-square tests for categorical variables. Effect sizes (eta-squared, η², for continuous variables and Cramer’s V for categorical variables) were calculated to characterize baseline equivalence.

The primary statistical approach for this study, defined prior to data analysis, comprised: (i) primary analysis: a 2 (group) × 3 (time) repeated-measures ANOVA for each outcome on participants completing all three assessments, with Greenhouse–Geisser correction applied when sphericity was violated. (ii) sensitivity analysis: a mixed-effects linear regression model with fixed effects for time, group, their interaction, age and sex, and a random intercept per participant, fitted on all randomized participants with at least one observation (intention-to-treat principle), under a missing-at-random assumption. The published trial protocol pre-specified an ANCOVA approach with baseline values as a covariate; we updated the analysis plan prior to data lock to better accommodate the three-time-point structure with partial missingness, in line with current best practice [4].

Within-group changes were further explored using paired-samples t-tests with 95% confidence intervals at each time-point contrast (pre–post, pre–retest, post–retest). Effect sizes were quantified using partial eta-squared (η²p) for time × group interactions (η²*p* ≥ 0.01 small, ≥ 0.06 medium, ≥ 0.14 large) and Cohen’s d for within-group changes (≤ 0.20 small, > 0.20 to ≤ 0.50 moderate, > 0.50 to ≤ 0.80 large, > 0.80 very large; [14, 24]).

To account for multiplicity, the family of three primary tests (2-AG, AEA, 6MWT) was controlled using the Holm–Bonferroni procedure at family-wise α = 0.05. Exploratory analyses (seven additional ES biomarkers and four additional muscular fitness outcomes) are reported with unadjusted p-values; their interpretation is restricted to hypothesis generation. All tests were two-sided, with α = 0.05. Statistical analyses were performed using STATA version 16.0 (Stata Corp, College Station, TX, USA).

## Results

### Participant flow and baseline characteristics

**Fig. 1:**
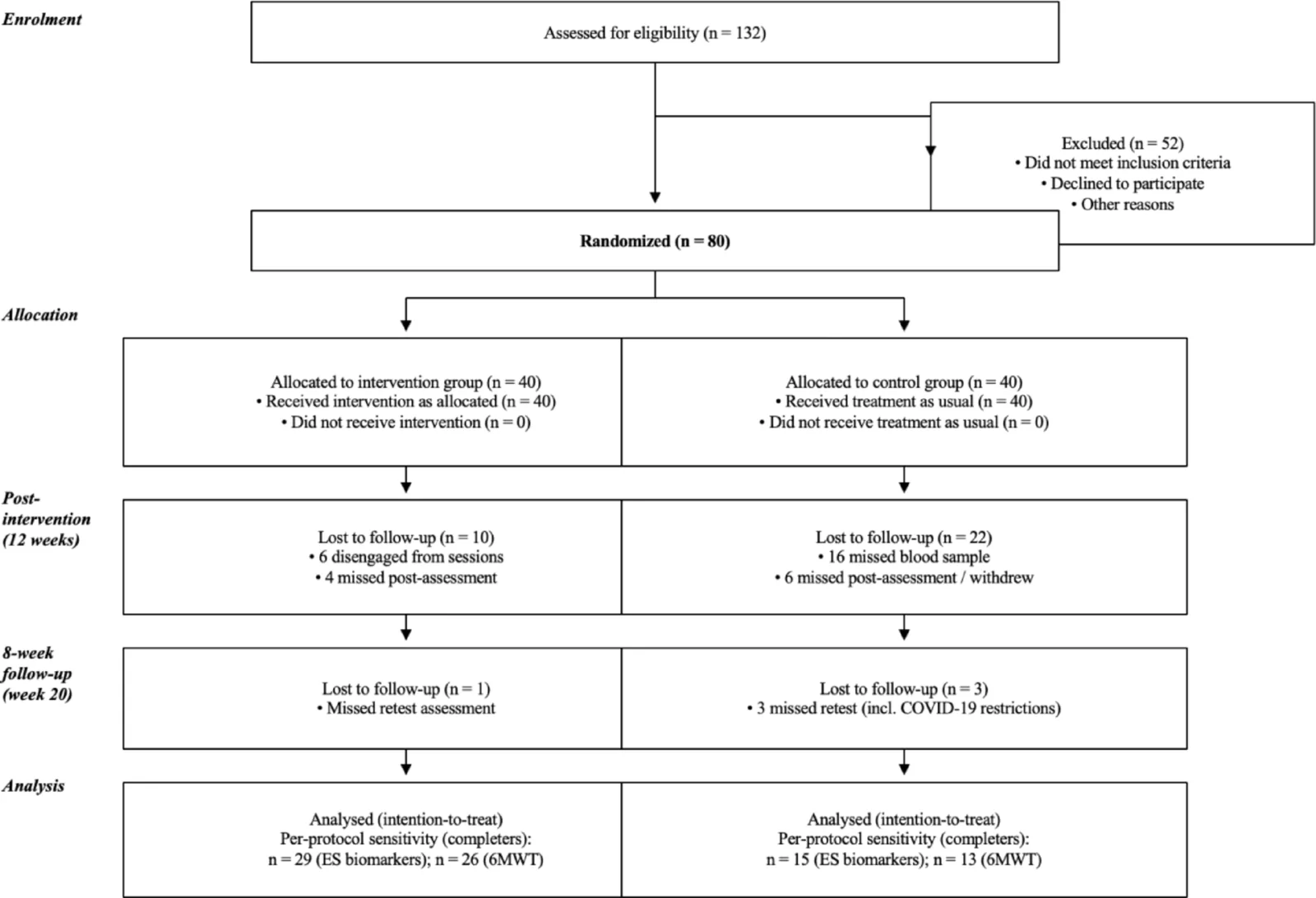
CONSORT 2025 participant flow diagram. Pre-specified outcomes: 2-AG, AEA, and 6-minute walking test (6MWT). The intention-to-treat primary analysis used a mixed-effects model on all randomized participants with at least one observation; the per-protocol sensitivity analysis used a 2 × 3 repeated-measures ANOVA on participants completing all three assessments

A CONSORT flow diagram is presented in Fig. 1. Eighty participants were randomly assigned to the IG (*n* = 40) or the CG (*n* = 40); attrition was substantial and asymmetric. By the end of the intervention, 29 participants in the IG and 15 in the CG completed all required assessments for both physical fitness measures and ES biomarkers (overall retention: 72.5% in the IG, 37.5% in the CG). Reasons for attrition (intervention discontinuation, withdrawal of consent, lost to follow-up, COVID-19-related restrictions during follow-up phases) are also summarized in Fig. 1. The differential dropout — substantially higher in the CG — is a major source of potential bias and is addressed in Limitations and through the intention-to-treat sensitivity analysis described above. Detailed information on the analytic sample size at each phase is summarized in Supplementary Table S1.

The demographic and baseline health characteristics — age, weight, BMI and lifestyle factors (smoking, alcohol consumption) — showed no significant differences between control and intervention groups, supporting effective randomization (all *p* > 0.05). Effect sizes were below thresholds for medium or large effects (Table 1).

**Table 1:** Descriptive characteristics of the study sample at baseline

Variable	*n*	All mean ± SD	Control mean ± SD	Intervention mean ± SD	F or χ²	*p*	Effect size η²
Age (years)	54	48.89 ± 10.23	49.43 ± 10.18	48.79 ± 10.42	0.05	0.82	0.01
Depression diagnosis (years)	49	1.88 ± 2.31	1.64 ± 1.88	2.03 ± 2.56	0.32	0.58	0.01
Weight (kg)	53	75.42 ± 17.39	74.43 ± 14.74	76.17 ± 19.38	0.13	0.72	0.01
Height (cm)	53	161.80 ± 7.93	163.29 ± 10.06	160.65 ± 5.71	1.46	0.23	0.03
BMI (kg/m²)	53	28.75 ± 6.20	27.80 ± 4.64	29.47 ± 7.17	0.94	0.33	0.02

Categorical variables	n	% of total	% in CG	% in IG	χ²	p	Cramer’s V
Sex	54				0.82	0.37	0.12
Women	47	87.0%	91.7%	83.3%			
Education level	53				3.97	0.41	0.27
Up to primary education	29	54.7%	52.2%	56.7%			
Higher education	24	45.3%	47.8%	43.3%			
Socioeconomic status	53				1.43	0.23	0.16
Easily	25	47.2%	56.5%	40.0%			
Difficultly	28	52.8%	43.5%	60.0%			
Cardiovascular disease	51				0.22	0.64	0.07
Yes	19	37.3%	40.9%	34.5%			
Diabetes	53				0.70	0.40	0.12
Yes	3	5.7%	8.7%	3.3%			
Arthritis	53				0.91	0.34	0.13
Yes	26	49.1%	56.5%	43.3%			
Cancer	51				< 0.001	0.95	0.01
Yes	16	31.4%	31.8%	31.0%			
Bodily pain interference	54				3.11	0.08	0.24
No to moderately	20	37.0%	50.0%	26.7%			
Quite to extremely	34	63.0%	50.0%	73.3%			
Marital status	53				0.09	0.76	0.04
Without couple	15	28.3%	30.4%	26.7%			
In a relationship	38	71.7%	69.6%	73.3%			
Smoking status	53				0.69	0.71	0.11
Never	25	47.2%	52.2%	43.3%			
Former	11	20.8%	21.7%	20.0%			
Current	17	32.1%	26.1%	36.7%			
Alcohol intake	52				4.09	0.13	0.28
Never	15	28.8%	34.8%	24.1%			
Former	11	21.2%	30.4%	13.8%			
Current	26	50.0%	34.8%	62.1%			
Antidepressant use	52				0.33	0.56	0.08
Yes	44	84.6%	94.7%	90.0%			

Continuous variables analyzed with one-way ANOVA. Categorical variables analyzed with chi-square test. Effect sizes: η² for continuous variables (≥ 0.01 small, ≥ 0.06 medium, ≥ 0.14 large); Cramer’s V for categorical variables (≥ 0.10 small, ≥ 0.30 medium, ≥ 0.50 large). All p values > 0.05

### Endocannabinoid system biomarkers

None of the time × group interactions for ES biomarkers reached statistical significance, including the primary biomarkers 2-AG (F(2,88) = 0.17, *p* = 0.844, η²*p* = 0.004) and AEA (F(2,88) = 1.20, *p* = 0.306, η²*p* = 0.027); results were unchanged after Holm–Bonferroni correction (Table 2).

**Table 2:** Mean (SD) of primary and exploratory outcomes across pre-, post-intervention and 8-week follow-up by group, with time × group interaction statistics from the primary repeated-measures ANOVA on completers

Outcome	Control group (*n* = 15)			Intervention group (*n* = 29)			F (T×G)	*p*	η²*p*	Holm-adj *p*	Cohen’s d (between, pre→post)	
	Pre	Post	Retest	Pre	Post	Retest						
*Primary endocannabinoid outcomes*												
2-AG (ng/mL)	2.60 (1.62)	**1.16 (0.50)** ^**^	**1.68 (0.88)†**	2.97 (1.73)	**2.35 (0.69)** ^***^	**1.90 (1.51)†**	0.17	0.844	0.004	0.844	−0.01	
AEA (ng/mL)	0.25 (0.11)	0.22 (0.09)	0.20 (0.07)	0.25 (0.09)	0.27 (0.11)	0.23 (0.09)	1.20	0.306	0.027	0.306	+ 0.42	
*Exploratory endocannabinoid analogues*												
OEA (ng/mL)	2.83 (1.06)	2.61 (0.81)	2.41 (0.81)	2.92 (0.70)	2.98 (0.91)	2.73 (0.84)	0.77	0.468	0.017	—	+ 0.31	
2-LG (ng/mL)	17.02 (13.51)	**6.26 (4.54)** ^**^	9.63 (5.99)	21.04 (16.40)	**5.49 (3.87)** ^***^	**11.98 (7.40)** ^††^	1.00	0.374	0.022	—	−0.23	
2-OG (ng/mL)	27.72 (18.11)	**14.31 (10.09)** ^*^	19.00 (10.74)	34.61 (26.95)	**12.51 (7.06)** ^***^	**21.73 (14.01)** ^†^	1.04	0.356	0.023	—	−0.29	
DEA (ng/mL)	0.07 (0.03)	0.06 (0.02)	**0.05 (0.02)** ^†^	0.07 (0.02)	0.07 (0.02)	0.06 (0.02)	0.38	0.683	0.009	—	+ 0.14	
DHEA (ng/mL)	0.37 (0.22)	0.29 (0.12)	0.31 (0.11)	0.35 (0.10)	0.34 (0.12)	0.33 (0.12)	2.50	0.087	0.054	—	+ 0.53	
LEA (ng/mL)	0.71 (0.35)	0.65 (0.27)	0.65 (0.27)	0.74 (0.15)	0.75 (0.23)	0.75 (0.23)	1.26	0.288	0.028	—	+ 0.56	
SEA (ng/mL)	1.29 (0.32)	1.39 (0.33)	1.40 (0.36)	1.37 (0.23)	1.49 (0.24)	1.42 (0.26)	1.22	0.300	0.027	—	+ 0.30	
*Primary cardiorespiratory fitness outcome*												
6MWT (m)	568.6 (88.0)	572.8 (63.7)	576.3 (75.8)	528.2 (70.8)	**609.9 (64.8)** ^***^	**583.1 (89.9)** ^†^	7.88	0.001	0.168	0.003	+ 1.27	
*Exploratory muscular fitness outcomes*												
Handgrip (kg)	25.3 (9.8)	23.3 (8.7)	25.5 (10.1)	24.6 (7.3)	25.7 (10.8)	25.8 (9.8)	0.29	0.747	0.007	—	+ 0.18	
Arm Curl (reps)	17.9 (4.6)	17.7 (3.9)	**15.8 (3.6)††**	17.4 (4.2)	17.4 (4.0)	17.9 (4.4)	2.83	0.065	0.068	—	+ 0.12	
Chair Stand (reps)	11.7 (2.7)	13.0 (2.4)	11.4 (2.5)	12.1 (2.9)	13.0 (2.7)	12.8 (2.4)	0.42	0.660	0.013	—	−0.18	
Standing Jump (cm) — < 45 y	88.6 (36.9)	83.7 (32.2)	92.3 (11.3)	85.3 (27.6)	**105.9 (26.4)** ^*^	103.9 (27.9)	0.93	0.402	0.033	—	+ 0.77	

Cohen’s d (between, pre→post) is the standardized between-group difference in the pre-to-post change, computed as (Δ_IG − Δ_CG) divided by the pooled SD of the change. Per-timepoint sample sizes are reported in Supplementary Table S1. Within-group post-hoc contrasts (mean differences, 95% confidence intervals and Cohen’s d for pre→post, pre→retest and post→retest) are reported in Supplementary Tables S2 (intervention group) and S3 (control group). The mixed-effects sensitivity analysis (intention-to-treat, all available data) is reported in Supplementary Table S4 and yielded concordant conclusions

*CG* Control group, *IG* Intervention group, *T × G* time × group interaction, *ng/mL* nanograms per millilitre, *reps* repetitions, *6MWT* 6-minute walking test

Within-group significance: post-intervention vs pre-intervention: *p < 0.05; **p < 0.01; ***p < 0.001. Retest vs pre-intervention: †p < 0.05; ††p < 0.01; †††p < 0.001. F, p and η²p refer to the time × group interaction from the primary repeated-measures ANOVA on participants completing all three assessments. Holm-adj p refers to the Holm–Bonferroni-corrected p-value across the three primary tests (2-AG, AEA and 6MWT); for exploratory outcomes the column shows ‘—’ because no within-family multiplicity correction was applied. Values in bold indicate statistically significant within-group change from pre-intervention

**Fig. 2:**
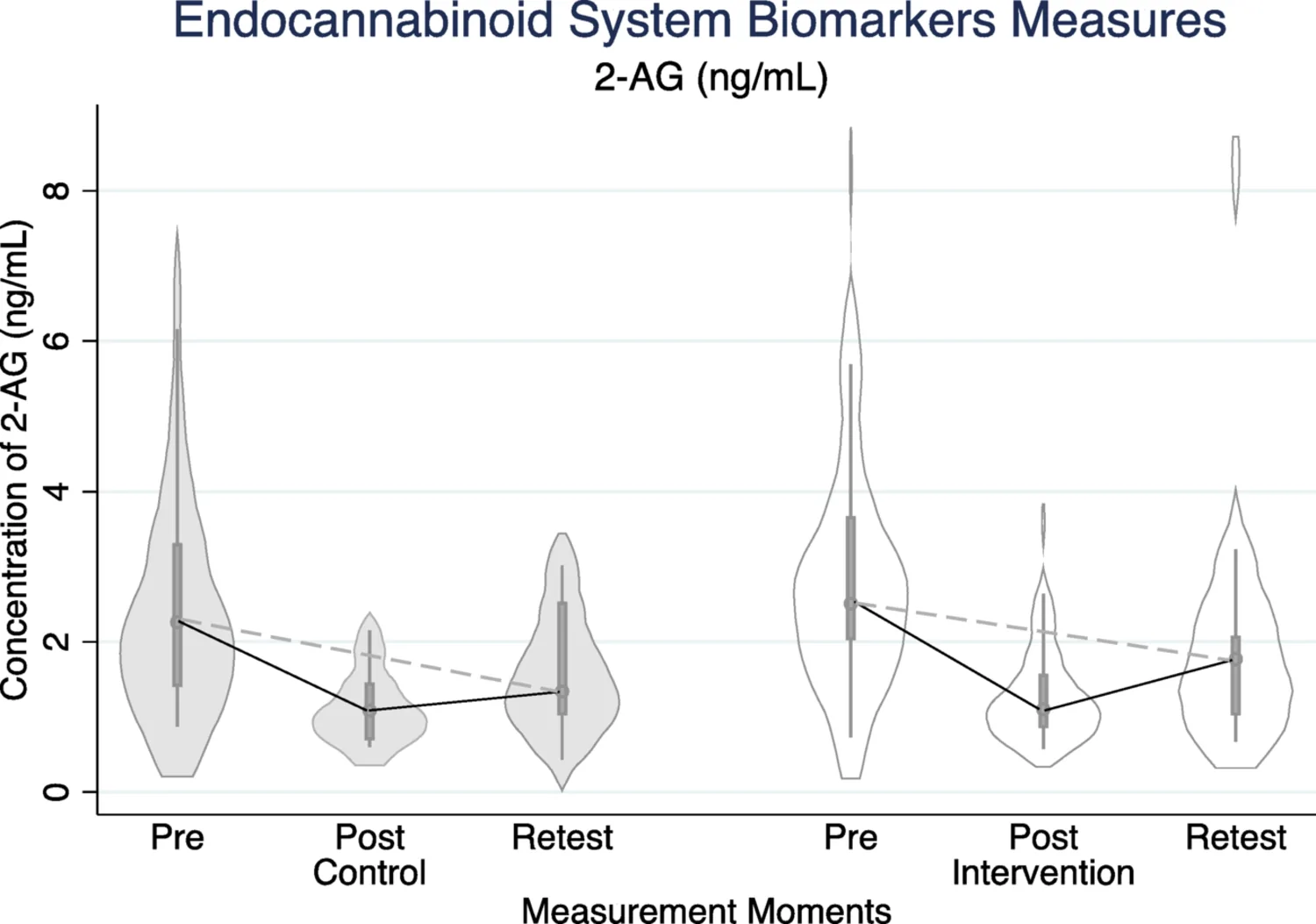
Plasma 2-AG concentrations across the three measurement points by group. Continuous lines connect group means at consecutive time points (pre-intervention, post-intervention at 12 weeks, 8-week follow-up); the dashed line indicates the pre-to-retest change. Within-group decreases were of comparable large magnitude in both groups (Supplementary Tables S2, S3); the time × group interaction was not statistically significant (F (2,88) = 0.17, *p* = 0.844, η²*p* = 0.004)

Within-group analyses showed parallel and large pre-to-post-intervention decreases in 2-AG in both groups (IG: −1.62 ng/mL [95% CI − 2.48 to − 0.76], *p* < 0.001; CG: −1.44 ng/mL [95% CI − 2.34 to − 0.53], *p* = 0.003), followed by partial rebound from post to retest in both groups (Supplementary Tables S2, S3). The magnitude of within-group change was almost identical between groups, consistent with the non-significant time × group interaction. Similar parallel decreases were observed for the analogues 2-LG and 2-OG (Table 2; with comparable large within-group decreases in both groups, all *p* < 0.05). Figure 2 illustrates 2-AG concentration changes across the three measurement moments for both groups.

AEA levels remained stable in both groups across the 12-week intervention period (within-group d ≤ 0.05 in IG and d = − 0.44 in CG, both *p* > 0.10). The exploratory ES analogues OEA, DEA, DHEA, LEA and SEA also showed no significant time × group interactions (all *p* > 0.15; Table 2).

The mixed-effects sensitivity analysis (intention-to-treat) yielded the same qualitative conclusions, i.e., no significant intervention effect on any ES biomarker (Supplementary Table S4).

### Physical fitness

**Fig. 3:**
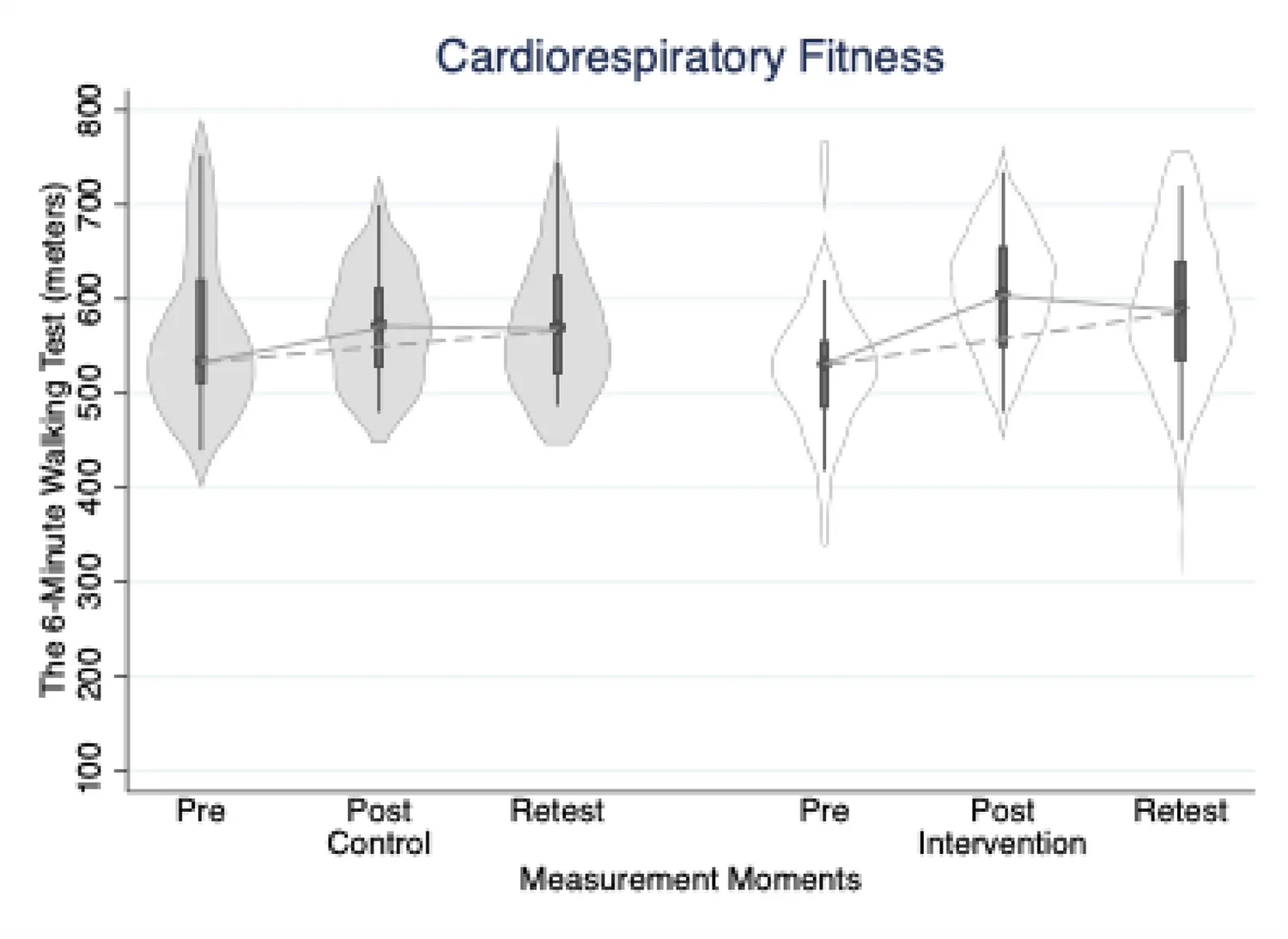
6-minute walking test distance (metres) across the three measurement points by group. Continuous lines connect group means at consecutive time points (pre-intervention, post-intervention at 12 weeks, 8-week follow-up); the dashed line indicates the pre-to-retest change. The time × group interaction was statistically significant (F(2,78) = 7.88, *p* = 0.001, η²*p* = 0.168, large effect) and survived Holm–Bonferroni correction across the three primary tests. The between-group difference in change at 12 weeks (≈ + 81 m) exceeds the 30–50 m minimal clinically important difference reported for the 6-minute walking test in clinical populations

The primary fitness outcome for this study — CRF assessed by the 6MWT — showed a significant time × group interaction (F(2,78) = 7.88, *p* = 0.001, η²*p* = 0.168, large effect; significant after Holm–Bonferroni correction across the three primary tests. Within the IG, distance covered increased by + 81.6 m at post-intervention (*p* < 0.001; full statistics in Supplementary Table S2) and remained + 54.8 m above baseline at retest (*p* < 0.05; Supplementary Table S2). The CG showed no significant change at any time point (Supplementary Table S3). The between-group difference in change at 12 weeks (+ 81.6 m) exceeds the 30–50 m minimal clinically important difference reported for the 6MWT in clinical populations [7]. Figure 3 displays these trajectories.

Among exploratory muscular fitness outcomes, no time × group interaction reached statistical significance. The arm curl test showed a borderline non-significant interaction (F(2,78) = 2.83, *p* = 0.065, η²*p* = 0.068), with a non-significant within-group decline in the CG at retest that was not mirrored in the IG (full within-group statistics in Supplementary Table S3); this exploratory pattern should be interpreted as hypothesis-generating only. Handgrip (F(2,72) = 0.29, *p* = 0.747, η²*p* = 0.007), chair stand (F(2,70) = 0.42, *p* = 0.660, η²*p* = 0.013) and standing jump (F(2,55) = 0.93, *p* = 0.402, η²*p* = 0.033) also did not show significant interactions. The standing jump analysis was severely underpowered (CG retest *n* = 5; jump was administered only to participants under 45 years of age) and should not be interpreted as evidence of a null effect; results are reported for completeness.

The mixed-effects sensitivity analysis yielded the same qualitative conclusions: a significant intervention effect on the 6MWT, and no significant effects on the four exploratory muscular fitness outcomes (handgrip, arm curl, chair stand, standing jump); see Supplementary Table S4.

## Discussion

**Fig. 4:**
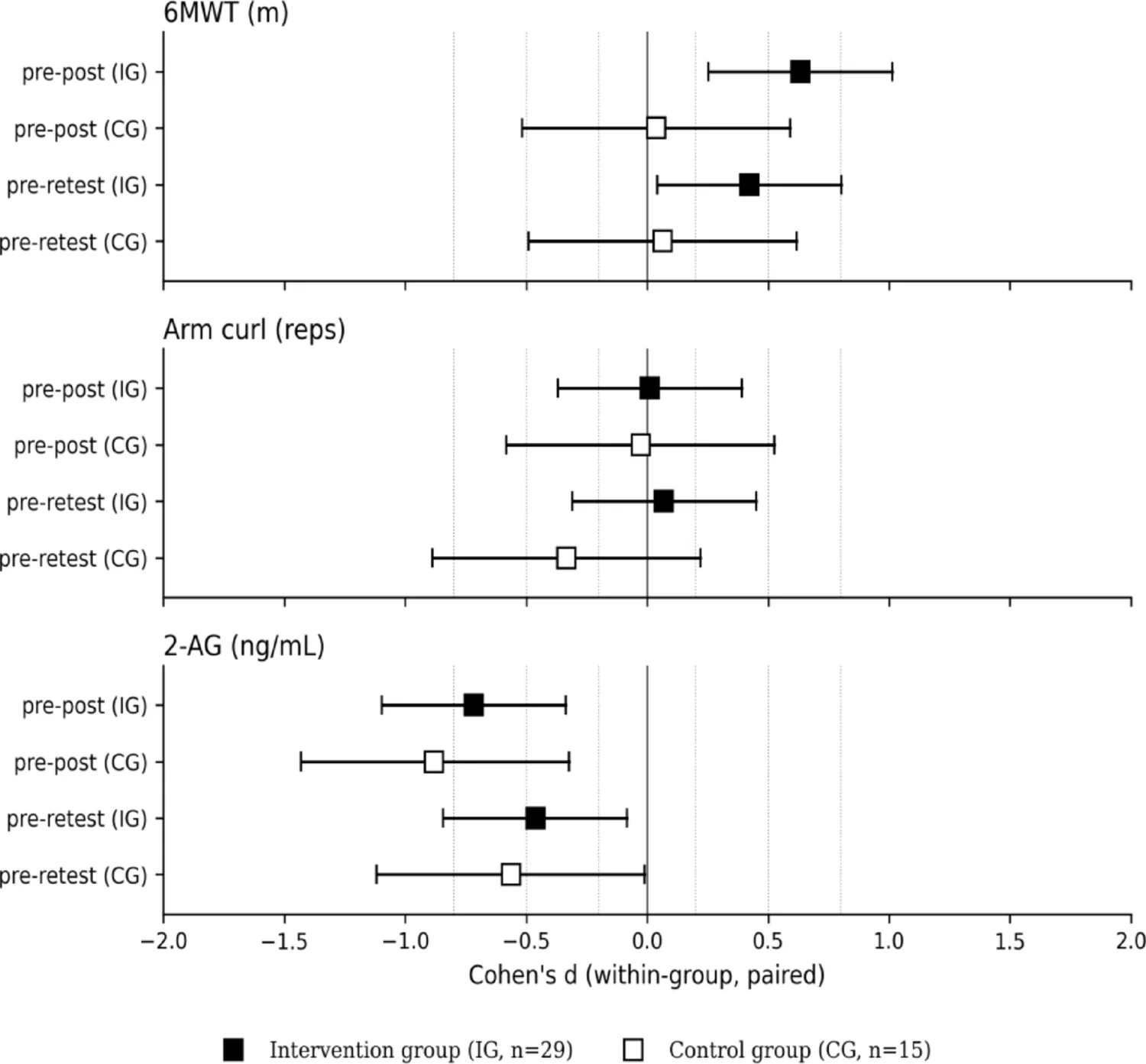
Forest plot of within-group standardized mean differences (Cohen’s d, 95% CI) for the primary outcomes (6MWT, 2-AG) and the arm curl test as exploratory illustration. Outcomes: 6-minute walking test (CRF), arm curl test, and 2-AG. Contrasts: pre-to-post and pre-to-retest, in the intervention group (filled squares) and the control group (open squares). Horizontal bars indicate 95% confidence intervals; vertical dotted lines mark conventional thresholds for small (|d| = 0.20), medium (|d| = 0.50) and large (|d| = 0.80) effects [14]. The 6-minute walking test shows the divergence between groups that drives the significant time × group interaction. 2-AG shows large parallel decreases in both groups, consistent with a non-specific time effect rather than an intervention-specific modulation. The arm curl test shows that the borderline non-significant interaction is driven by a within-group decline at retest in the control group rather than by gains in the intervention group

In this analysis of the SONRIE randomized controlled trial, a 12-week internet-based combined exercise and iCBT intervention significantly improved CRF in adults with mild-to-moderate depression compared with treatment as usual. The improvement was clinically meaningful (between-group difference at 12 weeks: +81.6 m on the 6MWT, exceeding the established minimal clinically important difference of 30–50 m) and partially preserved at the 8-week follow-up. A non-significant trend toward preservation of arm curl performance was also observed, with a within-group decline in the CG that was not mirrored in the IG; this exploratory pattern did not reach statistical significance and is reported as hypothesis-generating only. We did not find evidence of an intervention-specific effect on plasma ES biomarkers (including the primary biomarkers 2-AG and AEA) or on handgrip, chair stand, or standing jump performance (Fig. 4).

These results are consistent with the body of evidence showing that structured exercise improves CRF in clinical populations, including those with depression [12, 61, 63]. They extend that literature by demonstrating that comparable gains can be achieved through a primarily internet-delivered intervention, which is relevant to overcoming access barriers [59]. At the same time, our results do not support the hypothesis that the combined intervention modulates plasma ES concentrations beyond changes that occur in the control group.

### Endocannabinoid system biomarkers

Our investigation into ES biomarkers, specifically AEA and 2-AG and their analogues 2-LG and 2-OG, revealed nuanced responses over time. Both groups showed parallel and large within-group decreases in 2-AG, 2-LG and 2-OG from baseline to 12 weeks, with similar large magnitudes in both groups (Supplementary Tables S2, S3). The absence of a between-group difference, despite the magnitude of the temporal change, points to a non-specific time effect rather than an intervention-specific modulation. Possible contributors to this temporal effect include regression to the mean from elevated baseline values (potentially reflecting acute stress or dietary patterns at recruitment), seasonal or behavioral changes during the trial period (which overlapped with COVID-19 mobility restrictions during follow-up phases), or biological adaptations to repeated venipuncture and overnight fasting [5]. These observations contrast with reports of acute increases in 2-AG following exercise [20, 41].

Previous research on ES biomarkers, particularly AEA and 2-AG concentrations, has explored both acute [15–17, 29, 35, 41, 42, 44, 55, 65] and chronic physical exercise scenarios [3, 9, 27, 35, 51]. The chronic literature is heterogeneous: some studies report increased AEA after extended training in clinical populations, including fibromyalgia [66] and substance use disorders [9]; others find no change or decreases in healthy adults after prolonged interventions [28, 35]. Our null findings are most consistent with the latter group of studies. Several methodological considerations may explain this heterogeneity: timing of blood sampling relative to the last exercise bout, fasting status, exercise modality and intensity, and biological compartment (plasma versus brain) [3, 31, 32, 51].

The trial period overlapped partially with the COVID-19 pandemic, which restricted daily routines and physical activity, increased anxiety, stress and depression, and likely affected ES activity in both groups. Such non-specific stressors could plausibly produce parallel changes in both arms and obscure intervention-specific effects on the ES, as observed here.

### Physical fitness

The intervention produced a clinically meaningful improvement in CRF, with a between-group difference of approximately 81 m on the 6MWT at 12 weeks — substantially larger than the minimal clinically important difference of 30–50 m reported across clinical populations [7]. This effect is consistent with the broader exercise-in-depression literature [21, 47, 52, 63] and supports the feasibility of delivering exercise programs through primarily online platforms in this population [59]. Maintenance of approximately 75% of the gain at the 8-week post-intervention retest suggests that, although a structured program is required to drive the change, fitness gains do not vanish immediately upon program cessation.

In the muscular fitness domain, no time × group interaction reached statistical significance. A non-significant trend was observed for the arm curl test, with a within-group decline in the CG that was not mirrored in the IG; this pattern would be consistent with the intervention preserving upper-limb endurance against a background of depression-related deconditioning, but the finding is exploratory, did not reach statistical significance and should be replicated before drawing inferences. Handgrip and chair stand showed no significant changes, and the standing jump analysis was severely underpowered (analytic *n* = 21, restricted to participants under 45 years of age) and should not be considered conclusive. Patterns are broadly consistent with the literature, where improvements in muscular fitness following exercise programs are highly dependent on modality, intensity and adherence, and are typically less responsive than cardiorespiratory adaptations to mixed-modality programs of moderate intensity [46, 49].

The lack of baseline differences in physical health indicators and lifestyle factors strengthens the inferences drawn for the CRF outcome; nevertheless, differential attrition tempers the strength of the causal interpretation, as discussed below. Improvements in CRF may also contribute indirectly to improvements in depressive symptoms [68]. 

### Strengths and limitations

Several limitations warrant consideration. First, attrition was substantial and asymmetric (37.5% completers in the CG vs. 72.5% in the IG), partly attributable to COVID-19-related restrictions during follow-up phases and to the reduced engagement that frequently accompanies usual-care arms in non-pharmacological trials. Although we pre-specified a sensitivity analysis using mixed-effects models on all available data (intention-to-treat principle), the differential attrition introduces a risk of selection bias, particularly if CG participants who dropped out differed systematically from those who remained. The observed CRF gain should therefore be considered an upper-bound estimate of the true intervention effect. Second, the trial was originally powered to detect an effect on the primary outcome (depressive symptoms) and is underpowered to detect small or moderate intervention effects on ES biomarkers; non-significant results for these outcomes do not constitute evidence of equivalence. Third, multiplicity affects the interpretation of exploratory analyses (seven additional ES biomarkers and four additional muscular fitness outcomes); these are reported as hypothesis-generating only, and none of them reached statistical significance. Fourth, the published statistical analysis plan specified an ANCOVA approach with baseline covariates; we updated this prior to data lock to better accommodate the three-time-point structure with partial missingness. Fifth, plasma concentrations of ES ligands may not fully reflect functional activity within the central nervous system, as receptor sensitivity and expression were not assessed. Sixth, the study population was 87% female and almost exclusively Spanish, which limits generalizability across sex and across cultures. Seventh, the standing jump analysis was administered only to participants under 45 years of age and is underpowered (analytic *n* = 21). Finally, while the internet-based intervention mitigated geographical barriers, it may not be applicable to populations with limited access to technology or digital literacy.

Strengths include the rigorous RCT design, the integration of both physical and psychological components in a fully online format, the use of designated primary outcomes, the application of a sensitivity analysis using mixed-effects models on all available data (intention-to-treat principle), and a follow-up assessment to evaluate maintenance of effects. The combined exercise + iCBT internet-delivered approach is scalable and could inform clinical service design in mental health settings where access to traditional programs is limited.

We did not directly measure depressive symptoms in the present sub-study (the primary outcome of the SONRIE trial is reported separately). The observed improvement in CRF, together with prior evidence linking fitness gains to mood [48, 68], supports embedding exercise-based interventions in broader treatment strategies, especially given limitations on conventional face-to-face care and the persistent under-prescription of exercise in psychiatric practice [36, 50, 56, 57].

## Conclusion

In adults with mild-to-moderate depression, a 12-week internet-based combined exercise and iCBT intervention significantly improved CRF compared with treatment as usual, with a clinically meaningful between-group difference of approximately 81 m on the 6MWT at 12 weeks and partial maintenance at 8-week follow-up. We did not find evidence of an intervention-specific effect on plasma ES biomarkers, nor on exploratory muscular fitness outcomes; both groups showed parallel temporal changes in 2-AG, 2-LG and 2-OG, suggesting non-specific effects of time. These findings support the integration of internet-delivered exercise and psychological therapy into comprehensive depression-care pathways, while highlighting the need for adequately powered trials to clarify whether and how exercise interacts with the ES in clinical populations.

## Supplementary Information

Below is the link to the electronic supplementary material.

Supplementary Material 1

Supplementary Material 2

## Data Availability

De-identified data supporting the findings of this study are available from the corresponding author on reasonable request.
