# Supplementary material for: Effects of an internet-based combined exercise and cognitive-behavioral therapy intervention on endocannabinoid system biomarkers and physical fitness in adults with mild-to-moderate depression: a SONRIE randomized controlled trial
Source: Eur Arch Psychiatry Clin Neurosci. 2026 Aug 4;276(7):3701–14. doi: 10.1007/s00406-026-02330-x (PMC13562311; doi:10.1007/s00406-026-02330-x)
Supplement: Supplementary file 1 [file 406_2026_2330_MOESM1_ESM.docx]

**CONSORT 2025 Checklist**

*Effects of an Internet-Based Combined Exercise and Cognitive-Behavioral Therapy Intervention on Endocannabinoid System Biomarkers and Physical Fitness in Adults with Mild-to-Moderate Depression: a SONRIE Randomized Controlled Trial*

Trial registration: ClinicalTrials.gov NCT05849792

*Reference: Hopewell S et al. CONSORT 2025 statement: updated guideline for reporting randomized trials. Nature Medicine 31, 1776–1783 (2025).*

| **Item No** | **Section / Topic** | **Checklist item** | **Reported on page / section** |
| --- | --- | --- | --- |
| ***Title and abstract*** | | | |
| **1a** | *Title and structured abstract* | Identification as a randomized trial in the title. | Title: "...a SONRIE Randomized Controlled Trial". Page 1. |
| **1b** |  | Structured summary of trial design, methods, results and conclusions. | Abstract: structured Background, Methods, Results, Conclusion. Pages 2–3. |
| ***Open science*** | | | |
| **2** | *Trial registration* | Name of trial registry, identifying number (with URL) and date of registration. | Methods, Study design: "ClinicalTrials.gov NCT05849792; registered 6 May 2023". Page 6. Open Science Statements (page 22). |
| **3** | *Protocol and statistical analysis plan* | Where the trial protocol and statistical analysis plan can be accessed. | Methods, Study design: citing Escudier-Vázquez et al., 2025. Page 6. Open Science Statements (page 22). |
| **4** | *Data sharing* | Where and how the de-identified individual participant data, statistical code and any other materials can be accessed. | Open Science Statements, Data sharing: "available from the corresponding author on reasonable request, conditional on signed data-use agreement". Page 22. |
| **5a** | *Funding and conflicts of interest* | Sources of funding and other support, role of funders. | Open Science Statements, Funding: PI0068-2018, CIBERFES; funders had no role. Page 22. |
| **5b** |  | Financial and non-financial conflicts of interest of the manuscript authors. | Open Science Statements, Conflicts of interest: "no financial or non-financial conflicts of interest related to this work". Page 22. |
| ***Introduction*** | | | |
| **6** | *Background and rationale* | Scientific background and rationale. | Introduction. Pages 4–5. |
| **7** | *Objectives* | Specific objectives related to benefits and harms. | Introduction, last paragraph (page 5): pre-specified primary outcomes (2-AG, AEA, 6MWT) and exploratory outcomes; harms reported in Methods, Harms subsection (page 11). |
| ***Methods*** | | | |
| **8** | *Patient and public involvement* | Details of patient or public involvement in the design, conduct and reporting of the trial. | Methods, Patient and public involvement subsection (page 6): no patient/public involvement; intervention co-developed with clinical staff. |
| **9** | *Trial design* | Description of trial design including type of trial, allocation ratio and framework. | Methods, Study design (page 6): randomized controlled trial, parallel-group, 1:1 allocation, superiority framework. |
| **10** | *Changes to trial protocol* | Important changes to the trial after it commenced including any outcomes or analyses that were not pre-specified, with reason. | Methods, Statistical analysis (page 12): protocol pre-specified ANCOVA; updated to repeated-measures ANOVA prior to data lock to better accommodate three-time-point structure with partial missingness. |
| **11** | *Trial setting* | Settings and locations where the trial was conducted. | Methods, Study design (page 6): Mental Health Clinical Management Unit of Puerto Real, Cádiz, Spain. Conducted September 2020–December 2023. |
| **12a** | *Eligibility criteria* | Eligibility criteria for participants. | Methods, Eligibility criteria (page 7). |
| **12b** |  | Eligibility criteria for sites and individuals delivering the intervention. | Methods, Eligibility criteria (page 7): degree in Sciences of Physical Activity and Sport with prior clinical exercise experience; doctoral-level clinical psychologist with CBT and emotional regulation experience. |
| **13** | *Intervention and comparator* | Intervention and comparator with sufficient details to allow replication. | Methods, Procedure (page 8); Physical exercise intervention (page 8); Psychological intervention (page 9). Reported following CERT (Slade et al., 2016). |
| **14** | *Outcomes* | Pre-specified primary and secondary outcomes, including measurement variable, analysis metric and time point. | Methods, Outcomes (page 9): pre-specified primary 2-AG, AEA, 6MWT; exploratory analogues and muscular fitness measures; primary contrast is time × group interaction at 12 weeks. |
| **15** | *Harms* | How harms were defined and assessed. | Methods, Harms subsection (page 11): assessed non-systematically by self-report to the exercise specialist, with incident log. |
| **16a** | *Sample size* | How sample size was determined. | Methods, Sample size (page 12, lines 11–20): determined for the primary outcome of the SONRIE trial (depressive symptoms; BDI). Post-hoc detectable effect sizes for ES (Cohen's d ≈ 0.85) and CRF (d ≈ 0.95) reported. |
| **16b** |  | Explanation of any interim analyses and stopping guidelines. | Not applicable; no interim analyses or stopping rules. |
| **17a** | *Randomization — Sequence generation* | Who generated the random allocation sequence and the method used. | Methods, Participants and randomization (page 7): allocation sequence generated by the principal investigator using STATA 16.0; simple randomization. |
| **17b** |  | Type of randomization and details of any restriction. | Methods, Participants and randomization (page 7): simple randomization, 1:1 allocation, no stratification or blocking. |
| **18** | *Allocation concealment* | Mechanism used to implement the random allocation sequence. | Methods, Participants and randomization (page 7): allocation sequence prepared by PI before recruitment; allocations transmitted to field staff only after each participant's baseline assessment was completed. |
| **19** | *Implementation* | Whether personnel who enrolled and assigned participants had access to the random allocation sequence. | Methods, Participants and randomization (page 7): personnel performing baseline measurements did not know the upcoming allocation. |
| **20a** | *Blinding* | Who was blinded after assignment to interventions. | Methods, Participants and randomization (page 7): outcome assessors blinded at all time points; data de-identified before analysis. Participants and intervention deliverers unblinded due to nature of the intervention. |
| **20b** |  | If blinded, how blinding was achieved and description of similarity of interventions. | Methods, Participants and randomization (page 7): blinding of outcome assessors maintained by withholding allocation labels from staff conducting blood sampling and physical fitness testing. |
| **21a** | *Statistical methods* | Statistical methods used to compare groups for primary and secondary outcomes, including harms. | Methods, Statistical analysis (pages 12–13): primary analysis 2 × 3 repeated-measures ANOVA on completers; sensitivity mixed-effects ITT model; within-group paired t-tests with 95% CI; partial η²p and Cohen's d as effect sizes. |
| **21b** |  | Definition of who is included in each analysis, and in which group. | Methods, Statistical analysis (pages 12–13): primary analysis on participants completing all three assessments (per-protocol completers, n = 44 ES / n = 39 CRF); sensitivity on all randomized participants with at least one observation (intention-to-treat). |
| **21c** |  | How missing data were handled in the analysis. | Methods, Statistical analysis (page 13): mixed-effects model accommodates missing data under MAR assumption; per-protocol completers analysis includes only participants with complete data. |
| **21d** |  | Methods for any additional analyses (subgroup, sensitivity), distinguishing pre-specified from post hoc. | Methods, Statistical analysis (page 13): mixed-effects ITT pre-specified as sensitivity analysis; Holm–Bonferroni correction on three pre-specified primary tests; exploratory analyses unadjusted. |
| ***Results*** | | | |
| **22a** | *Participant flow, including flow diagram* | Numbers of participants randomly assigned, received intended intervention and analysed for the primary outcome, by group. | Results, Participant flow and baseline characteristics (page 14); Figure 1 (CONSORT flow diagram, separate editable file Figure1_CONSORT_flow.pptx). |
| **22b** |  | Losses and exclusions after randomization, with reasons, by group. | Results, Participant flow and baseline characteristics (page 14); Figure 1. |
| **23a** | *Recruitment* | Dates defining the periods of recruitment and follow-up. | Methods, Study design (page 6): trial conducted between September 2020 and December 2023. |
| **23b** |  | If relevant, why the trial ended or was stopped. | Not applicable; trial completed as planned. |
| **24a** | *Intervention and comparator delivery* | Intervention and comparator as actually administered (who delivered, adherence, fidelity). | Methods, Physical exercise intervention (pages 8–9): exercise specialist with prior clinical experience; supported by physical therapist during first four weeks; achieved adherence in IG = 72.5% (median 28/36 sessions, IQR 24–32). Methods, Psychological intervention (page 9): doctoral-level clinical psychologist. |
| **24b** |  | Concomitant care received during the trial for each group. | Methods, Procedure (page 8, lines 1–6): CG received treatment as usual at the Mental Health Clinical Management Unit; no structured exercise prescription, iCBT or additional contact from the research team beyond the three scheduled assessments. |
| **25** | *Baseline data* | Table showing baseline demographic and clinical characteristics for each group. | Table 1. |
| **26** | *Numbers analyzed, outcomes and estimation* | For each primary and secondary outcome, by group: number of participants included in the analysis, number with available data at each time point, results, estimated effect size and precision. | Table 2; Supplementary Table S1 (per-timepoint sample sizes); Supplementary Tables S2 and S3 (within-group post-hoc with mean differences, 95% CI and Cohen's d categorical bins); Supplementary Table S4 (sensitivity ITT mixed-effects). |
| **27** | *Harms* | All harms or unintended events in each group. | Methods, Harms (page 11): no serious adverse events; minor self-limited muscle soreness reported by 6 (15%) IG participants in the first two weeks; no adverse events in CG. |
| **28** | *Ancillary analyses* | Any other analyses performed, including subgroup and sensitivity analyses, distinguishing pre-specified from post hoc. | Methods, Statistical analysis (page 13); Results: pre-specified primary analysis (RM-ANOVA on completers); pre-specified sensitivity (mixed-effects ITT, Supplementary Table S4); pre-specified Holm–Bonferroni correction on three primary tests (2-AG, AEA, 6MWT). |
| ***Discussion*** | | | |
| **29** | *Interpretation* | Interpretation consistent with results, balancing benefits and harms, and considering other relevant evidence. | Discussion (pages 16–21): main interpretation focuses on the significant CRF improvement; ES findings interpreted as non-significant intervention effect against background of parallel temporal change in both groups; arm curl interpreted as a non-significant trend (borderline interaction p = 0.065), hypothesis-generating only. |
| **30** | *Limitations* | Trial limitations, addressing sources of potential bias, imprecision, generalizability and multiplicity of analyses. | Discussion, Strengths and limitations (pages 19–21): differential attrition; underpowered for ES; multiplicity (none of the exploratory tests reached statistical significance); deviation from protocol-pre-specified ANCOVA; plasma vs CNS; female-predominant sample; underpowered standing-jump subgroup. |

This checklist follows the CONSORT 2025 statement for reporting randomized trials (Hopewell S et al., Nature Medicine 31, 1776–1783, 2025). Page references correspond to the revised manuscript. Item numbering and wording adhere to the CONSORT 2025 expanded checklist.
