# Supplementary material for: Effects of an internet-based combined exercise and cognitive-behavioral therapy intervention on endocannabinoid system biomarkers and physical fitness in adults with mild-to-moderate depression: a SONRIE randomized controlled trial
Source: Eur Arch Psychiatry Clin Neurosci. 2026 Aug 4;276(7):3701–14. doi: 10.1007/s00406-026-02330-x (PMC13562311; doi:10.1007/s00406-026-02330-x)
Supplement: Supplementary file 2 [file 406_2026_2330_MOESM2_ESM.docx]

| **Table S1:** Number of participants with available data in each main study variable, by group and time point. | | | | | | | |
| --- | --- | --- | --- | --- | --- | --- | --- |
|  | **Control Group** | | | **Intervention Group** | | | |
|  | **Pre** | **Post** | **Retest** | **Pre** | **Post** | **Retest** |  |
| **Endocannabinoid system biomarkers** | 24 | 18 | 14 | 30 | 30 | 29 |  |
| (2-AG, AEA, OEA, 2-LG, 2-OG, DEA, DHEA, LEA, SEA) |  |  |  |  |  |  |  |
| **Physical Fitness** | |  |  |  |  |  |  |
| Cardiorespiratory Fitness | |  |  |  |  |  |  |
| *6MWT* | 24 | 16 | 13 | 30 | 27 | 26 |  |
| **Muscular Fitness** | |  |  |  |  |  |  |
| *Handgrip* | 24 | 17 | 15 | 30 | 29 | 25 |  |
| *Arm Curl* | 23 | 17 | 14 | 29 | 28 | 24 |  |
| *Chair stand* | 23 | 15 | 14 | 30 | 29 | 26 |  |
| *Jump* | 21 | 13 | 6 | 26 | 25 | 21 |  |

CG, control group (40 randomized); IG, intervention group (40 randomized). Numbers shown are participants with a non-missing value at each time point. The strict analytic database used for the per-protocol primary analysis comprises participants with at least a baseline endocannabinoid measurement (n = 54: 24 CG + 30 IG).

**Table S2.** Post-hoc test statistics for within-group changes (pre-to-post, pre-to-retest, post-to-retest) in the intervention group (IG, n = 29 for endocannabinoid biomarkers; n = 24 for the 6-minute walking test; complete cases).

| **Outcome** | **Pre→Post mean diff. (95% CI)** | **Pre→Retest mean diff. (95% CI)** | **Post→Retest mean diff. (95% CI)** | **d P→Po** | **d P→R** | **d Po→R** |
| --- | --- | --- | --- | --- | --- | --- |
| ***Endocannabinoid system biomarkers*** | | | | | | |
| 2-AG (ng/mL) | **-1.62*** (-2.48, -0.76)** | **-1.05* (-1.92, -0.19)** | 0.56 (-0.30, 1.43) | +++ | ++ | ++ |
| AEA (ng/mL) | 0.01 (-0.05, 0.07) | -0.02 (-0.09, 0.04) | -0.03 (-0.10, 0.03) | - | + | + |
| OEA (ng/mL) | 0.06 (-0.46, 0.58) | -0.25 (-0.71, 0.33) | -0.25 (-0.77, 0.27) | - | + | + |
| 2-LG (ng/mL) | **-15.55*** (-22.27, -8.83)** | **-9.06** (-15.84, -2.29)** | 6.49 (-0.29, 13.27) | +++ | ++ | +++ |
| 2-OG (ng/mL) | **-22.10*** (-33.48, -10.73)** | **-12.89* (-24.36, -1.41)** | 9.22 (-2.26, 20.69) | +++ | ++ | +++ |
| DEA (ng/mL) | -0.00 (-0.02, 0.01) | -0.01 (-0.02, 0.01) | -0.00 (-0.02, 0.01) | - | + | - |
| DHEA (ng/mL) | -0.02 (-0.09, 0.05) | -0.03 (-0.10, 0.05) | -0.01 (-0.08, 0.06) | - | + | - |
| LEA (ng/mL) | 0.02 (-0.11, 0.14) | -0.07 (-0.19, 0.06) | -0.08 (-0.21, 0.04) | - | + | + |
| SEA (ng/mL) | 0.12 (-0.03, 0.27) | 0.05 (-0.11, 0.20) | 0.05 (-0.11, 0.20) | ++ | - | + |
| ***Physical fitness*** | | | | | | |
| 6MWT (m) | **81.64*** (32.63, 130.64)** | **54.84* (5.34, 104.34)** | -26.79 (-77.55, 23.97) | +++ | ++ | + |
| Handgrip (kg) | 1.06 (-4.90, 7.01) | 1.19 (-5.00, 7.37) | 0.13 (-6.11, 6.37) | - | - | - |
| Arm Curl (reps) | 0.07 (-2.64, 2.78) | 0.51 (-2.31, 3.34) | 0.45 (-2.40, 3.29) | - | - | - |
| Chair Stand (reps) | 0.87 (-0.83, 2.56) | 0.67 (-1.08, 2.42) | -0.20 (-1.96, 1.57) | + | + | - |
| Standing Jump (cm) | **20.59* (1.83, 39.35)** | **18.56 (-1.09, 38.21)** | -2.02 (-21.85, 17.80) | ++ | ++ | - |

Mean differences are computed from paired-samples t-tests within the intervention group (complete-case sample). Significance: post-intervention vs pre-intervention: *p < 0.05; **p < 0.01; ***p < 0.001. Retest vs pre-intervention: †p < 0.05; ††p < 0.01; †††p < 0.001. Cohen's d is reported for all contrasts; values are interpreted as |d| ≤ 0.20 small, > 0.20 to ≤ 0.50 moderate, > 0.50 to ≤ 0.80 large, > 0.80 very large (Cohen, 1992). d P→Po, Cohen's d for pre-to-post; d P→R, pre-to-retest; d Po→R, post-to-retest.

**Table S3.** Post-hoc test statistics for within-group changes (pre-to-post, pre-to-retest, post-to-retest) in the control group (CG, n = 15 for endocannabinoid biomarkers; n = 12 for the 6-minute walking test; complete cases).

| **Outcome** | **Pre→Post mean diff. (95% CI)** | **Pre→Retest mean diff. (95% CI)** | **Post→Retest mean diff.**  **(95% CI)** | **d P→Po** | **d P→R** | **d Po→R** |
| --- | --- | --- | --- | --- | --- | --- |
| ***Endocannabinoid system biomarkers*** | | | | | | |
| 2-AG (ng/mL) | **-1.44** (-2.34, -0.53)** | **-0.97* (-1.93, -0.02)** | 0.46 (-0.55, 1.48) | +++ | ++ | ++ |
| AEA (ng/mL) | -0.03 (-0.10, 0.05) | **-0.05 (-0.13, 0.02)** | -0.03 (-0.11, 0.05) | + | ++ | + |
| OEA (ng/mL) | -0.22 (-0.93, 0.50) | -0.42 (-1.17, 0.34) | -0.20 (-1.00, 0.60) | + | + | + |
| 2-LG (ng/mL) | **-10.76** (-18.21, -3.31)** | **-7.39 (-15.26, 0.48)** | 3.37 (-4.98, 11.73) | +++ | ++ | ++ |
| 2-OG (ng/mL) | **-13.41* (-24.36, -2.47)** | -8.72 (-20.27, 2.83) | 4.69 (-7.57, 16.96) | +++ | ++ | + |
| DEA (ng/mL) | -0.01 (-0.03, 0.00) | **-0.02* (-0.04, -0.00)** | -0.00 (-0.02, 0.01) | ++ | +++ | + |
| DHEA (ng/mL) | -0.08 (-0.21, 0.04) | -0.06 (-0.20, 0.08) | 0.02 (-0.12, 0.17) | + | + | + |
| LEA (ng/mL) | -0.06 (-0.30, 0.17) | **-0.14 (-0.38, 0.11)** | -0.07 (-0.33, 0.19) | + | + | + |
| SEA (ng/mL) | 0.10 (-0.15, 0.36) | 0.11 (-0.16, 0.38) | 0.01 (-0.28, 0.29) | + | + | - |
| ***Physical fitness*** | | | | | | |
| 6MWT (m) | 4.14 (-58.58, 66.87) | 7.70 (-59.23, 74.63) | 3.56 (-69.01, 76.13) | - | - | - |
| Handgrip (kg) | -1.96 (-9.45, 5.53) | 0.27 (-7.51, 8.05) | 2.23 (-6.14, 10.61) | - | - | - |
| Arm Curl (reps) | -0.16 (-3.42, 3.09) | **-2.08 (-5.54, 1.37)** | -1.92 (-5.60, 1.75) | - | + | ++ |
| Chair Stand (reps) | **1.35 (-0.76, 3.46)** | -0.22 (-2.38, 1.93) | -1.57 (-3.94, 0.79) | ++ | - | ++ |
| Standing Jump (cm) | -4.88 (-34.08, 24.32) | 3.76 (-34.54, 42.06) | 8.64 (-32.19, 49.48) | - | - | + |

Mean differences are computed from paired-samples t-tests within the control group (complete-case sample). Significance: post-intervention vs pre-intervention: *p < 0.05; **p < 0.01; ***p < 0.001. Retest vs pre-intervention: †p < 0.05; ††p < 0.01; †††p < 0.001. Cohen's d is reported for all contrasts; values are interpreted as |d| ≤ 0.20 small, > 0.20 to ≤ 0.50 moderate, > 0.50 to ≤ 0.80 large, > 0.80 very large (Cohen, 1992). d P→Po, Cohen's d for pre-to-post; d P→R, pre-to-retest; d Po→R, post-to-retest.

**Table S4.** Sensitivity analysis — comparison of the primary 2 × 3 repeated-measures ANOVA (per-protocol completers) and the mixed-effects intention-to-treat model for the time × group interaction.

| **Outcome** | **RM-ANOVA (primary) F, p, η²p** | **Mixed-effects ITT (sensitivity)** | **Conclusion** |
| --- | --- | --- | --- |
| 2-AG (ng/mL) | F(2,88) = 0.17, p = 0.844, η²p = 0.004 | Concordant — non-significant | Time × group interaction not significant in either analysis. |
| AEA (ng/mL) | F(2,88) = 1.20, p = 0.306, η²p = 0.027 | Concordant — non-significant | Time × group interaction not significant in either analysis. |
| 6MWT (m) | F(2,78) = 7.88, p = 0.001, η²p = 0.168 | Concordant — significant | Robust to choice of analysis; survives Holm–Bonferroni correction across primary tests. |
| Arm curl (reps) | F(2,78) = 2.83, p = 0.065, η²p = 0.068 | Concordant — non-significant | Exploratory; non-significant in both analyses; should be replicated. |

The 2 × 3 repeated-measures ANOVA on participants completing all three assessments is the primary analysis reported in the main manuscript (Table 2); the mixed-effects intention-to-treat model fitted with fixed effects for time × group, age and sex, and a random intercept per participant on all randomized participants with at least one observation is reported here as a sensitivity analysis.
